# Health-Related Quality of Life and Mid-Term Oncological Outcomes After Subcostal Uniportal Robot-Assisted Lung Cancer Surgery: A Secondary Analysis of a Clinical Trial

**DOI:** 10.3390/cancers18172763

**Published:** 2026-08-25

**Authors:** Chuan Cheng, Ya-Chun Hsu, Yin-Kai Chao

**Affiliations:** 1 Division of Thoracic Surgery, New Taipei Municipal Tu-Cheng Hospital, New Taipei City 236017, Taiwan; cheng_c134569@hotmail.com; 2 Department of Artificial Intelligence, Graduate Institute of Clinical Medical Sciences, Chang Gung University, Taoyuan 333323, Taiwan; 3 Division of Thoracic Surgery, Chang Gung Memorial Hospital, College of Medicine, Chang Gung University, Taoyuan 333423, Taiwan; 4 Department of Nursing, Chang Gung Memorial Hospital-Linkou, Chang Gung University, Taoyuan 333423, Taiwan

**Keywords:** anatomical lung resection, uniportal robotic surgery, subcostal approach, chronic pain, neuropathic pain, health-related quality of life

## Abstract

Objective: Subcostal uniportal robot-assisted thoracic surgery (URATS) using the da Vinci Single-Port (SP) system avoids intercostal placement of the main robotic access. This prespecified secondary analysis of a prospective trial characterized chronic postsurgical pain and health-related quality of life (HRQOL) as primary patient-reported outcomes after subcostal URATS for early-stage lung cancer, with mid-term oncological outcomes as an exploratory endpoint. Methods: Patients from the parent trial (NCT05535712) undergoing subcostal URATS were assessed at 3, 6, and 12 months by structured telephone interviews. Chronic postsurgical pain was defined by the ICD-11 criterion (Numerical Rating Scale [NRS] > 0 on any of four activity-specific items). Neuropathic features were assessed with the painDETECT Questionnaire (PDQ), and HRQOL with EORTC QLQ-C30 summary score and selected domains. Results: In 33 patients, chronic postsurgical pain declined from 33.3% at 3 months to 6.1% at 12 months (*p* = 0.004), was mild among symptomatic patients (median NRS 1), and showed no PDQ-defined neuropathic features. Two patients received a 7-day course of gabapentin at 3 months. Baseline HRQOL (median QLQ-C30 summary score 91.5) increased at all postoperative time points (*p* < 0.001). At a median 40-month follow-up, one patient had bilateral pulmonary recurrence at 13 months, all remained alive, and no delayed hernia or other access-related complication was identified. Conclusions: Subcostal URATS was feasible in this prospective cohort. Chronic postsurgical pain was mild and decreased over time, HRQOL remained well preserved, although data on mid-term oncological outcomes were preliminary.

## Simple Summary

Conventional lung cancer surgery is typically performed through the intercostal space and may result in chronic postsurgical pain due to intercostal nerve injury. In this secondary analysis of a prospective clinical trial, we evaluated mid-term pain, health-related quality of life (HRQOL), and oncological outcomes after subcostal uniportal robot-assisted thoracic surgery (URATS) using the da Vinci SP system for early-stage lung cancer. Among 33 patients, chronic pain progressively decreased during follow-up and remained consistently mild, with no neuropathic pain features detected. HRQOL remained high throughout follow-up. After a median follow-up of 40 months, the 3-year overall survival and disease-free survival rates were 100% and 96.9%, respectively, although these estimates should be interpreted cautiously given the small number of events. These prospective data provide an initial description of mid-term patient-reported outcomes after subcostal URATS. However, larger comparative studies are required to determine whether avoiding placement of the main robotic access through an intercostal space improves postoperative pain or quality of life and to confirm oncological outcomes.

## 1. Introduction

Minimally invasive approaches, including video-assisted thoracoscopic surgery (VATS) and robot-assisted thoracic surgery (RATS), have increasingly replaced thoracotomy for anatomical lung resection. Compared with thoracotomy, minimally invasive approaches are associated with less surgical trauma and improved postoperative recovery [1,2,3,4]. Although these approaches reduce surgical trauma and facilitate postoperative recovery, intercostal access may still injure the intercostal neurovascular bundle and contribute to chronic postsurgical pain [5,6]. Chronic pain has been reported in 6.9–42.0% of patients after conventional VATS or RATS [1,7,8,9,10,11].

The subcostal thoracic approach avoids placement of the main surgical access through an intercostal space and may therefore reduce direct contact with the intercostal neurovascular bundle [12,13,14]. When attempted via conventional VATS or multiport robotic platforms, the subcostal technique has historically been limited by restricted hilar exposure [15]. However, the introduction of the da Vinci SP system (Intuitive Surgical Inc., Sunnyvale, CA, USA)—a single-port platform featuring three independently articulating instruments and an advanced stereoscopic wristed camera—has enabled the development of subcostal uniportal robot-assisted thoracic surgery (URATS) [12]. Early clinical series have demonstrated the technical feasibility and acceptable short-term postoperative outcomes of subcostal URATS with the da Vinci SP system [16,17,18,19,20,21], but evidence beyond the immediate postoperative period remains limited [22]. In particular, longitudinal data on chronic postsurgical pain, health-related quality of life (HRQOL), and oncological outcomes are lacking. Therefore, in this prespecified secondary analysis of a prospective clinical trial, we aimed to characterize chronic postsurgical pain and HRQOL as primary patient-reported outcomes after subcostal URATS, with mid-term oncological outcomes as an exploratory endpoint.

## 2. Materials and Methods

### 2.1. Study Design

This is a prespecified secondary analysis of a prospective clinical trial (ClinicalTrials.gov identifier: NCT05535712). The study protocol was published previously [18], and detailed inclusion and exclusion criteria are provided in Supplementary Table S1. Briefly, eligible patients were 20–75 years old, had clinical stage I lung cancer with a tumor measuring ≤4 cm and located at least 2 cm from the lobar bronchial origin, and were considered suitable for anatomical lung resection. Patients requiring extended resection or reconstruction, with a history of ipsilateral thoracic surgery or neoadjuvant treatment, with major cardiopulmonary contraindications, or with anatomy considered unsuitable for minimally invasive surgery were not eligible for enrollment in the original trial. Body mass index and tumor location did not affect eligibility or enrollment in the trial. Lobectomy was considered the standard anatomical resection. Segmentectomy was considered for tumors < 2 cm when an adequate surgical margin, defined as >2 cm or greater than twice the tumor diameter, was considered achievable based on preoperative imaging and three-dimensional reconstruction of computed tomography images. All procedures were performed by a single surgeon who had completed more than 500 multiport robotic thoracic procedures and had received formal da Vinci SP cadaveric training before initiation of the trial. The surgeon had also performed 10 subxiphoid da Vinci SP procedures for anterior mediastinal lesions, as described in the original trial report.

Participants in the original trial were recruited consecutively between November 2022 and May 2023. The study protocol adhered to the ethical guidelines established by the Declaration of Helsinki and received approval from the Institutional Review Board at Chang Gung Memorial Hospital-Linkou, Taiwan (reference number: CGMH-IRB 202101423A0) on 12 October 2021. Written informed consent was obtained from each participant prior to enrollment. Clinical and perioperative data were extracted from medical records.

For the present secondary analysis, patients who required conversion to thoracotomy during the index procedure or subsequently underwent ipsilateral surgery through an intercostal approach during follow-up were excluded to minimize the potential influence of additional chest wall trauma on the assessment of pain and HRQOL following the subcostal approach. Postoperative pain intensity, pain location, qualitative pain characteristics, neuropathic pain features, and HRQOL were assessed through structured telephone interviews conducted by the same research assistant using standardized questionnaires, including the Numerical Rating Scale (NRS), painDETECT Questionnaire (PDQ), and European Organization for Research and Treatment of Cancer Quality of Life Questionnaire (EORTC QLQ-C30).

### 2.2. Subcostal URATS Surgical Procedure

**Figure 1:**
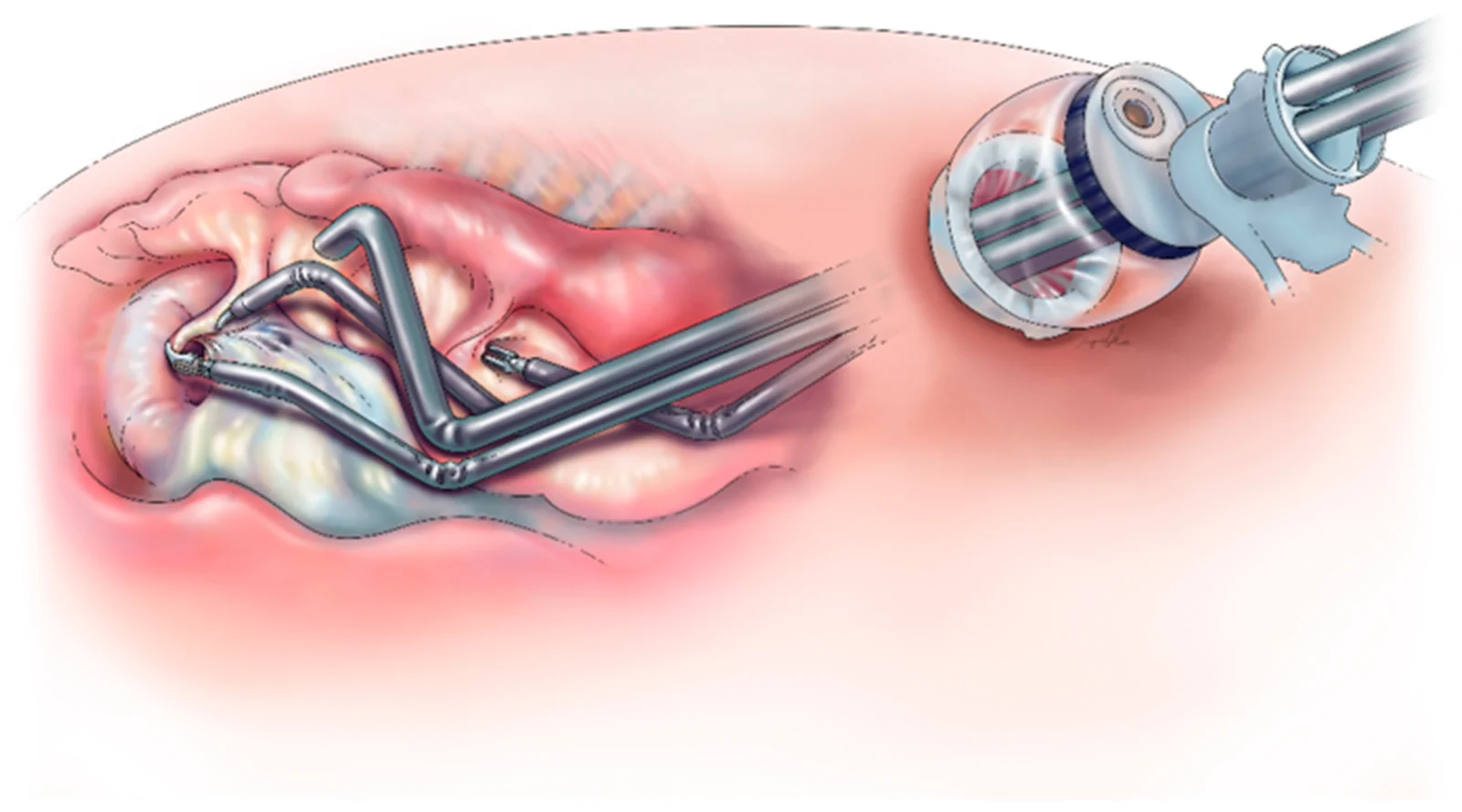
Operative configuration for subcostal URATS. The robotic arms are introduced through a single subcostal incision without intercostal spreading, allowing access to the thoracic cavity while avoiding placement of the main robotic access through an intercostal space.

Following the induction of general anesthesia, patients were placed in the lateral decubitus position. A 10-mm intercostal observation port was used when required for thoracoscopic guidance during creation of the subcostal access. A 4-cm incision was created at the intersection of the subcostal arch and mid-clavicular line. Subcutaneous tissue and oblique muscles were dissected until the transversus abdominis fascia was exposed. Access to the pleural space was achieved by tunneling beneath the costal cartilage and above the diaphragm using blunt finger dissection and electrocauterization [12,21]. A uniportal access device (da Vinci SP Access Port Kit, large incision; Intuitive Surgical, Inc., Sunnyvale, CA, USA) was inserted and connected to an insufflator. The large SP Access Port was then docked to the da Vinci SP patient-side cart arm, facilitating the subsequent procedure (Figure 1). The operation followed standard console surgeon techniques for lung resection using the multi-arm robotic platform. Robotic instruments, including monopolar curved scissors, Maryland bipolar forceps, fenestrated bipolar forceps, and Cadiere forceps, were used for hilar and mediastinal lymph node dissection. Target pulmonary vessels were encircled with vessel loops and dissected sufficiently to permit stapler insertion. A handheld endovascular stapling instrument was inserted through the assistant port of the da Vinci SP Access Port Kit via the subcostal incision. For left-sided procedures, particular attention was paid to instrument angulation and shaft positioning to minimize interference with the beating heart. Upon procedure completion, the diaphragm edge was reattached to the transverse abdominis fascia using previously placed surgical ropes. A curved chest tube was inserted through the subcostal incision before final wound closure [17,18].

### 2.3. Perioperative Pain Management

Perioperative analgesia was administered according to routine clinical practice. At the completion of surgery, 200 mg of ropivacaine was infiltrated into the chest wall incision sites followed by intercostal nerve block administration. During emergence from anesthesia, patients were given intravenous fentanyl or a long-acting nonsteroidal anti-inflammatory drug (NSAID) when clinically indicated. Postoperative analgesia was based primarily on non-opioid regimens, including acetaminophen, NSAIDs, or tramadol/acetaminophen (Ultracet^®^), tailored to individual tolerance and comorbidities. Additional analgesics such as morphine and parecoxib were administered when clinically indicated.

### 2.4. Study Endpoints

The primary endpoint of the original trial was intraoperative conversion, whereas secondary endpoints included perioperative results, postoperative pain, and HRQOL. The primary endpoint and 30-day perioperative outcomes, including early postsurgical pain, have been previously reported [18]. This secondary analysis therefore focused on chronic postsurgical pain and HRQOL at 3, 6, and 12 months. Recurrence and survival outcomes were also assessed during oncological follow-up.

### 2.5. Outcome Definitions

Postoperative pain intensity was assessed using NRS [8]. Assessment included four activity-specific items: pain at rest, pain while walking, pain during physical activity, and pain intensity during the most recent episode of worst pain. Each item was scored from 0 (no pain) to 10 (worst imaginable pain). Chronic postsurgical pain was defined according to ICD-11 as pain localized to the surgical area persisting for ≥3 months after surgery [23]. At each follow-up assessment, any chronic postsurgical pain was defined as an NRS score greater than 0 on at least one of the four items, provided that the pain was localized to the surgical area. For descriptive analysis, the highest score across the four NRS items was defined as the maximum NRS at each time point. In this study, “any postsurgical pain” denotes the prevalence of pain (NRS > 0 on at least one item), whereas “Pain intensity” indicates the NRS score. Analgesic treatment and PDQ-defined neuropathic features were reported separately as additional indicators of the clinical burden of pain. The PDQ—which was administered to identify neuropathic components [24,25]—comprises three components: pain gradation, pattern of pain course, and radiating pain. The pain gradation component evaluates seven pathologic pain sensations (burning, tingling/pricking, tactile and thermal allodynia, electric shock-like sensations, numbness, and pressure-evoked pain), with each sensation graded from 0 (none) to 5 (very strongly), yielding scores from 0 to 35. The pattern of pain course component is scored as 0 (persistent pain with slight fluctuations), −1 (persistent pain with pain attacks), 1 (pain attacks without pain between them), or 1 (pain attacks with pain between them). The radiating pain component is scored as either 2 or 0, depending on whether pain radiates to other body regions. The total PDQ score (range: −1 to 38) categorizes pain as likely neuropathic (≥19), unlikely neuropathic (≤12), or uncertain (13–18).

HRQOL was concurrently assessed using the EORTC QLQ-C30 [26,27,28,29]. All scale scores were linearly transformed to a 0–100 range. Higher functional-scale and global health status scores indicate better functioning or quality of life, whereas higher symptom-scale scores indicate greater symptom burden. The QLQ-C30 summary score was calculated as the mean of 13 scales after reversing the symptom scales, such that a higher summary score represented better overall HRQOL.

Pain and HRQOL assessments were performed for all patients included in the present secondary analysis, regardless of final pathological diagnosis. In contrast, oncological analyses were restricted to the 32 patients with a final pathological diagnosis of lung cancer. Overall survival (OS) was calculated from the date of surgery to death from any cause, and disease-free survival (DFS) from the date of surgery to the first documented recurrence or death. Patients without an event were censored at the date of last follow-up.

### 2.6. Data Analysis

Categorical variables are presented as frequencies and percentages. Continuous variables are summarized as mean with standard deviation or median with range, as appropriate. Repeated binary outcomes across the 3-, 6-, and 12-month assessments were compared using Cochran’s Q test. Repeated ordinal or non-normally distributed continuous outcomes were compared using the Friedman test. When an overall test was statistically significant, pairwise comparisons were performed using McNemar tests or Wilcoxon signed-rank tests, as appropriate, with Holm adjustment for multiple comparisons [30]. NRS and PDQ scores among patients reporting pain were summarized descriptively because the composition of this subgroup differed across follow-up assessments. A two-sided *p* value < 0.05 was considered statistically significant. Survival probabilities were estimated using the Kaplan–Meier method, with 95% confidence intervals (CIs) reported for the 3-year OS and DFS rates. All statistical analyses were performed using SPSS Statistics, version 20.0 (IBM Corp., Armonk, NY, USA).

## 3. Results

### 3.1. Patient Population

A total of 35 patients were initially enrolled in the trial. Two patients were subsequently excluded—one who required intraoperative conversion to thoracotomy and one who underwent reoperation via an intercostal approach—leaving 33 patients for the analysis of chronic pain and HRQOL. All 33 patients completed follow-up. Table 1 summarizes patient and tumor characteristics. One patient (3.0%) was upstaged from clinical N0 to pathological N2 disease and was classified as pT2aN2M0, stage IIIA.

### 3.2. Chronic Pain and HRQOL

**Figure 2:**
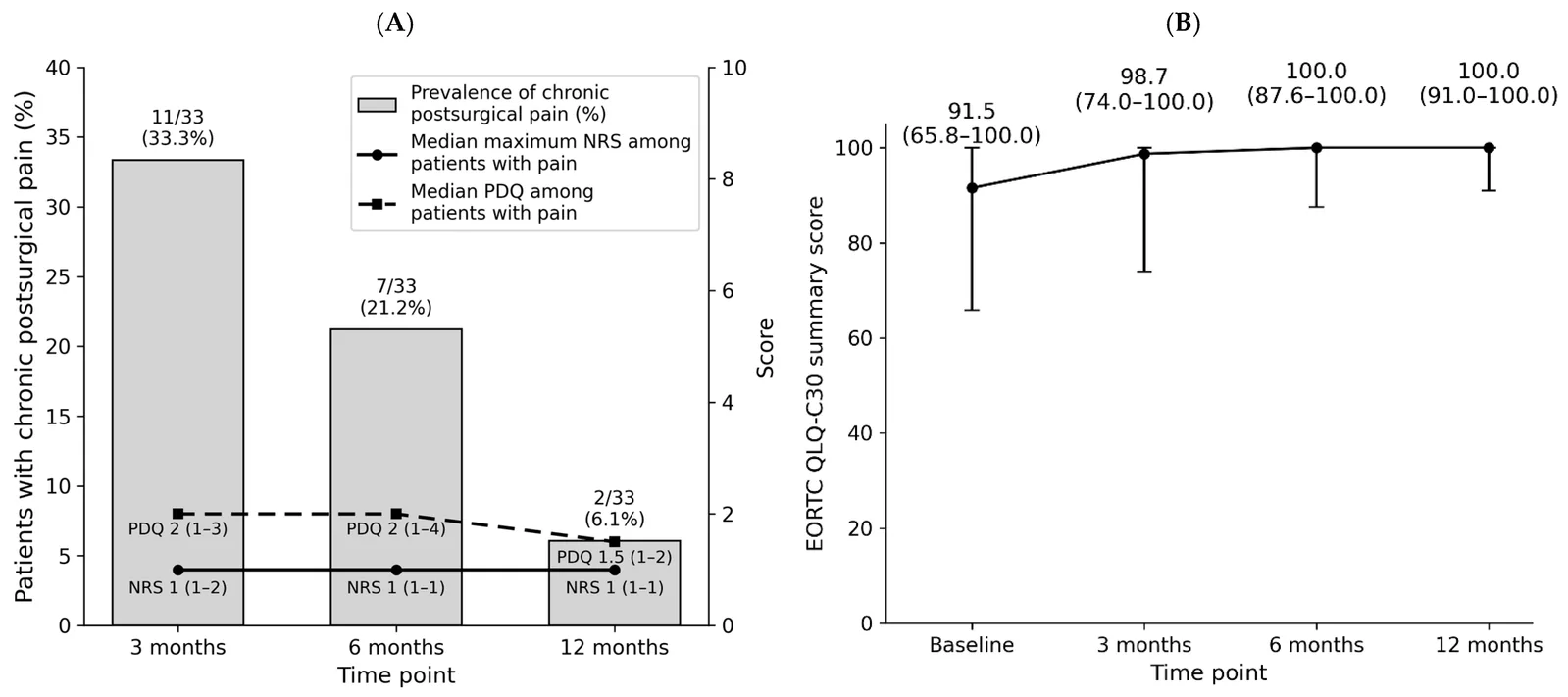
(**A**) Prevalence and severity of any postsurgical pain at 3, 6, and 12 months postoperatively. Bar graph showing the number of patients reporting any postsurgical pain (NRS > 0) at each time point (left y-axis). Median maximum NRS and PDQ scores among symptomatic patients are shown as solid and dashed lines, respectively (right y-axis). (**B**) EORTC QLQ-C30 summary scores at baseline and at 3, 6, and 12 months postoperatively. Higher scores reflect better health-related quality of life.

Table 2 and Figure 2A summarize chronic pain outcomes after subcostal URATS. Among the 33 patients, any postsurgical pain (NRS > 0) was reported by 11 patients (33.3%) at 3 months, 7 (21.2%) at 6 months, and 2 (6.1%) at 12 months. The prevalence differed significantly over time (*p* = 0.004), with a significant reduction from 3 to 12 months after adjustment for multiple comparisons (adjusted *p* = 0.012). All reported pain was localized to the surgical area, primarily the subcostal incision site.

Pain intensity was mild across assessments, with a maximum NRS of 2 and a median NRS of 1 among patients reporting pain at each assessment. Maximum NRS scores in the entire cohort decreased significantly over time (*p* = 0.004), with a significant reduction from 3 to 12 months after adjustment for multiple comparisons (adjusted *p* = 0.012) (Supplementary Table S2).

PDQ scores were all ≤ 12, within the “neuropathic pain unlikely” category. Only two patients required analgesic treatment for postsurgical pain, both at the 3-month follow-up (7-day course of gabapentin); no additional analgesic treatment was required during subsequent follow-up, and no long-term analgesic use was documented. During the 40-month follow-up, no delayed incisional hernia at the subcostal site, wound complications, or other access-related complications were identified.

HRQOL outcomes are summarized in Table 2 and Figure 2B. The median EORTC QLQ-C30 summary score increased from 91.5 (range, 65.8–100.0) at baseline to 98.7 (74.0–100.0) at 3 months and 100.0 at both 6 months (87.6–100.0) and 12 months (91.0–100.0; *p* < 0.001). Scores were significantly higher at all postoperative assessments than at baseline. The score was also significantly higher at 6 months than at 3 months (adjusted *p* = 0.012), whereas no significant difference was observed between 6 and 12 months.

Among the individual EORTC QLQ-C30 domains, global health status/QoL, pain, and fatigue improved significantly during follow-up. Physical functioning showed an overall difference across assessments, although no pairwise comparison remained significant after adjustment, while role functioning and dyspnea remained stable (Supplementary Table S3).

### 3.3. Exploratory Mid-Term Oncological Outcomes

**Figure 3:**
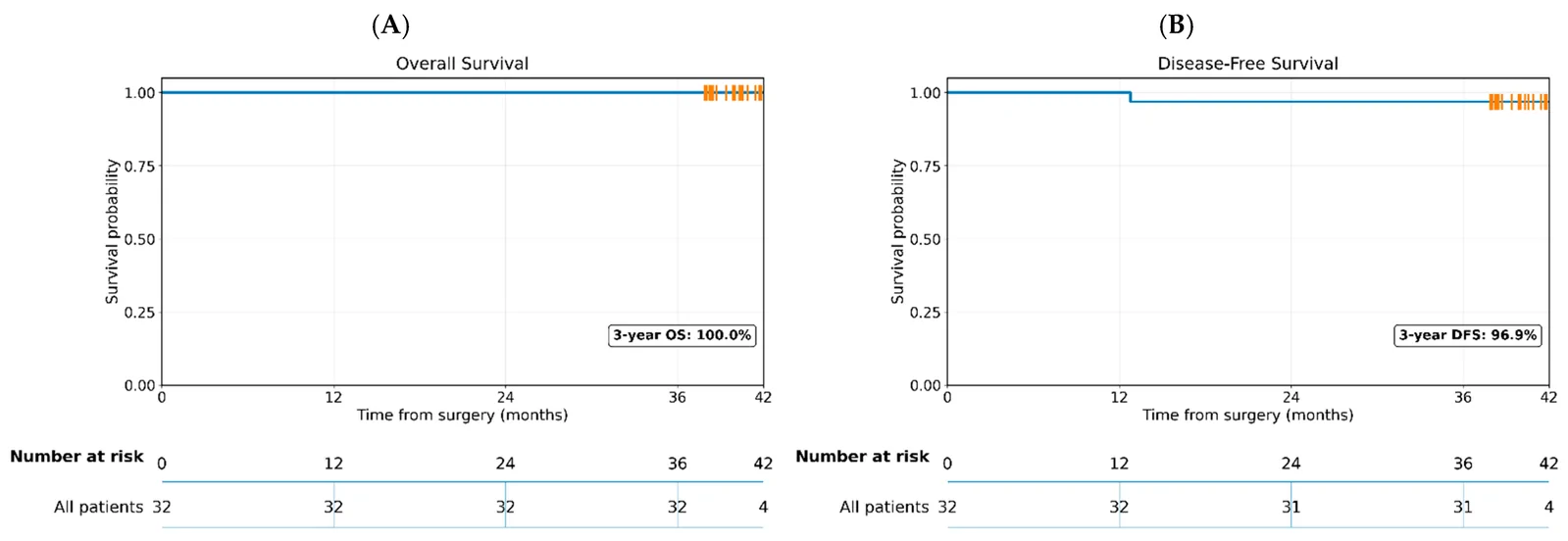
Kaplan–Meier survival curves for the 32 patients included in the oncological analysis. (**A**) Overall survival (OS): 3-year rate 100%. (**B**) Disease-free survival (DFS): 3-year rate 96.9%. Blue step lines represent Kaplan–Meier survival estimates, and orange vertical tick marks indicate censored observations. Numbers at risk are shown below each panel; vertical tick marks indicate censored observations. These estimates are exploratory, given the limited number of events during follow-up.

Among the 32 patients with lung cancer, eight (25.0%) received postoperative adjuvant therapy, which was completed in seven. After a median follow-up of 40 months, all patients remained alive, and one patient developed bilateral pulmonary recurrence 13 months after surgery. The 3-year OS rate (Figure 3A) and DFS rate (Figure 3B) were 100.0% (95% confidence interval (CI), 100.0–100.0%) and 96.9% (95% CI, 79.8–99.6%), respectively.

## 4. Discussion

In this prespecified secondary analysis of a prospective clinical trial, we characterized the longitudinal patterns of chronic postsurgical pain, HRQOL, and mid-term oncological outcomes after subcostal URATS using the da Vinci SP system. Any postsurgical pain, defined by the sensitive ICD-11 criterion of an NRS score > 0, was reported by 33.3% of patients at 3 months and progressively decreased to 6.1% at 12 months. The discrepancy between a 33.3% prevalence of any pain at 3 months and a consistently low median maximum NRS of 1 among symptomatic patients indicates that prevalence alone may overstate the clinical burden of postsurgical pain. PDQ assessment showed no features consistent with a neuropathic component throughout follow-up; this finding is reported descriptively as the absence of PDQ-defined neuropathic pain features in the studied cohort rather than as evidence that neuropathic mechanisms were definitively absent. HRQOL, which was already high at baseline (median EORTC QLQ-C30 summary score 91.5), increased significantly at all postoperative assessments and remained near the ceiling of the scale throughout follow-up. The mid-term oncological outcomes should be interpreted as exploratory, given that only one recurrence and no deaths were observed during a median follow-up of 40 months. No delayed access-related complication was identified during follow-up; early perioperative complications (30-day) were prospectively collected in the parent trial and are reported in the original trial publication [18]. Together, these prospective longitudinal observations are intended as a clinical reference for the design of future comparative studies, including randomized trials.

The observed decline in prevalence indicates that estimates of chronic postsurgical pain are highly dependent on the timing of assessment. Future randomized comparisons of subcostal and intercostal SP approaches should incorporate multidimensional pain assessment, including pain prevalence, pain intensity (NRS), neuropathic pain features (PDQ), analgesic requirements, and patient-reported HRQOL. The longitudinal event rates and score distributions observed in the present cohort may provide preliminary estimates to inform endpoint selection, assessment timing, and sample-size planning for future randomized trials.

This study has several limitations. First, this was a small, single-center secondary analysis without a concurrent intercostal RATS or VATS comparator, precluding conclusions regarding comparative pain, HRQOL, or oncological outcomes. Second, the exclusion of the two patients who underwent conversion to thoracotomy or subsequent ipsilateral intercostal surgery introduced post-enrollment selection bias; because both patients had additional intercostal chest wall trauma, this exclusion likely biased the observed pain and HRQOL outcomes toward more favorable estimates. Third, all procedures were performed by an experienced robotic thoracic surgeon at a high-volume center, and the original trial used relatively restrictive eligibility criteria; both factors may limit the external validity of these findings. A formal learning-curve threshold for subcostal URATS with the da Vinci SP system cannot be estimated from a single-operator cohort of this size, and reproducibility of these outcomes in less specialized settings will need to be assessed in future multi-center studies. Patients with more advanced disease, higher comorbidity burden, or prior thoracic surgery or neoadjuvant treatment were not eligible for the parent trial and may show different pain, HRQOL, or oncological trajectories that cannot be inferred from the present cohort. Fourth, quantitative opioid-equivalent analgesic consumption was not prospectively collected and could not be reconstructed reliably from the medical records; pain scores alone therefore cannot be interpreted as evidence of reduced postoperative pain burden. Fifth, three pain-measurement caveats should be noted. The chronic-pain definition of NRS > 0 was chosen as a sensitive threshold aligned with the ICD-11 definition, but it may overestimate the prevalence of clinically meaningful pain. The small sample and the telephone-based administration of the questionnaires by a single research assistant may also have introduced a reporting bias. Baseline NRS and PDQ scores were not collected in the parent trial, so preoperative-to-postoperative changes in pain intensity and neuropathic pain features cannot be estimated. Finally, the ceiling effect observed with the EORTC QLQ-C30, which is expected in the relatively fit early-stage cohort selected by the trial’s restrictive eligibility criteria, and the small number of oncological events limit the interpretation of HRQOL and survival estimates.

## 5. Conclusions

Subcostal URATS with the da Vinci SP system was feasible in this single-arm prospective cohort. Postsurgical pain was generally mild and progressively decreased over 12 months, with no PDQ-defined neuropathic pain features and well-preserved HRQOL. Mid-term oncological outcomes were preliminary. These findings are presented as promising early results requiring confirmation in larger comparative studies, and may provide a useful reference for designing future randomized trials comparing subcostal and intercostal SP approaches.

## Figures and Tables

**Table 1:** Clinicopathological characteristics of the 33 patients included in the secondary analysis.

Variable	Value
Age, years	60.12 (9.34)
Sex	
Men	8 (24.2%)
Women	25 (75.8%)
Body mass index, kg/m^2^	24.41 (4.55)
Charlson comorbidity index	
0	25 (75.8%)
1	5 (15.2%)
≥2	3 (9.1%)
Tumor size, cm	1.8 (0.9)
Tumor location	
Right upper lobe	10 (30.3%)
Right middle lobe	7 (21.2%)
Right lower lobe	8 (24.2%)
Left upper lobe	2 (6.1%)
Left lower lobe	6 (18.2%)
Type of surgery	
Lobectomy	28 (84.8%)
Segmentectomy	5 (15.2%) *
Complete resection	33 (100%)
Harvested nodal stations	6 (4–8)
Harvested lymph nodes	13 (5–37)
Histology	
Atypical adenomatous hyperplasia/adenocarcinoma in situ	2 (6.1%)
Invasive adenocarcinoma	28 (84.8%)
Carcinoid tumor	2 (6.1%)
Sclerosing pneumocytoma	1 (3.0%)
Pathological stage of malignant lung tumors (*n* = 32)	
Stage 0 (includes premalignant tumor)	2 (6.3%)
Stage IA1	9 (28.1%)
Stage IA2	9 (28.1%)
Stage IA3	4 (12.5%)
Stage IB	7 (21.9%)
Stage IIIA	1 (3.1%)
Pathological nodal status (*n* = 32)	
pN0	31 (96.9%)
pN1	0 (0.0%)
pN2	1 (3.1%)
Adjuvant treatment (*n* = 32)	8 (25.0%)

Continuous variables are presented as mean (standard deviation) or median (range), as appropriate, and categorical variables as number (percentage). Pathological staging was determined according to the eighth edition of the American Joint Committee on Cancer staging system. Detailed perioperative outcomes of the original clinical trial have been reported previously. Pathological stage, pathological nodal status, and adjuvant treatment percentages are based on the 32 patients included in the oncological analysis; the single patient with sclerosing pneumocytoma was excluded because this lesion is not a malignant lung tumor. * The five segmentectomies comprised one left lingulectomy, two left superior segmentectomies, one left anterior basal segmentectomy and one right posterior segmentectomy.

**Table 2:** Prevalence and characteristics of chronic pain and quality of life at 3, 6, and 12 months following subcostal uniportal robot-assisted thoracic surgery.

Variable	3 Months	6 Months	12 Months
Patients with postsurgical pain, *n* (%)	11 (33.3%)	7 (21.2%)	2 (6.1%)
Maximum NRS, entire cohort	0 (0–2)	0 (0–1)	0 (0–1)
Maximum NRS among patients reporting pain	1 (1–2)	1 (1–1)	1 (1–1)
PDQ score among patients reporting pain	2 (1–3)	2 (1–4)	1.5 (1–2)
PDQ classification			
Neuropathic-likely	0	0	0
Uncertain	0	0	0
Neuropathic-unlikely	11 (100%)	7 (100%)	2 (100%)
Patients receiving analgesic treatment for chronic pain, *n* (%)	2 (6.1%)	0 (0.0%)	0 (0.0%)
EORTC QLQ-C30 summary score, entire cohort	98.7 (74.0–100.0)	100 (87.6–100.0)	100 (91.0–100.0)

Continuous variables are presented as median (range), and categorical variables as number (percentage). “Patients with postsurgical pain” refers to any postsurgical pain (NRS > 0 on at least one of the four pain items), following the ICD-11 definition; “Patients receiving analgesic treatment for chronic pain” indicates patients who required pharmacologic treatment for persistent postsurgical pain. Abbreviations: EORTC QLQ, European Organization for Research and Treatment of Cancer Quality of Life Questionnaire; NRS, Numerical Rating Scale; PDQ, painDETECT Questionnaire.

## Data Availability

The data that support the findings of this study are available from the corresponding author upon reasonable request.

## Supplementary Materials

The following supporting information can be downloaded at: https://www.mdpi.com/article/10.3390/cancers18172763/s1, Supplementary Table S1. Inclusion and exclusion criteria. Supplementary Table S2. Activity-specific and episode-specific pain outcomes at 3, 6, and 12 months. Supplementary Table S3. Longitudinal EORTC QLQ-C30 summary and selected domain scores.
