# Supplementary material for: Health-Related Quality of Life and Mid-Term Oncological Outcomes After Subcostal Uniportal Robot-Assisted Lung Cancer Surgery: A Secondary Analysis of a Clinical Trial
Source: Cancers (Basel). 2026 Aug 25;18(17):2763. doi: 10.3390/cancers18172763 (PMC13565155; doi:10.3390/cancers18172763)
Supplement: Supplementary file 1 [file cancers-18-02763-s001.zip › 20260820SUPPLEMENTARY_TABLE_S3.pdf]

Supplementary Table S3. Longitudinal EORTC QLQ-C30 summary and selected domain scores

**EORTC QLQ-C30 scale**	**Baseline**	**3 months**	**6 months**	**12 months**	**Overall *p* value**
Global health status/QoL	66.7 (0–100.0)	83.3 (66.7–100.0)	100.0 (50.0–100.0)	100.0 (66.7–100.0)	< 0.001
Physical functioning	100.0 (60.0–100.0)	100.0 (73.3–100.0)	100.0 (66.7–100.0)	100.0 (66.7–100.0)	0.009
Role functioning	100.0 (66.7–100.0)	100.0 (83.3–100.0)	100.0 (66.7–100.0)	100.0 (66.7–100.0)	0.355
Pain	0 (0–50.0)	0 (0–50.0)	0 (0–33.3)	0 (0–0)	< 0.001
Fatigue	11.1 (0–44.4)	0 (0–33.3)	0 (0–22.2)	0 (0–33.3)	< 0.001
Dyspnea	0 (0–33.3)	0 (0–100.0)	0 (0–33.3)	0 (0–33.3)	0.141
**Summary score**	**91.5 (65.8–100.0)**	**98.7 (74.0–100.0)**	**100.0 (87.6–100.0)**	**100.0 (91.0–100.0)**	**< 0.001**

Values are presented as median (range). Overall comparisons across baseline and the 3-, 6-, and 12-month assessments were performed using the Friedman test. When the overall test was statistically significant, pairwise comparisons were performed using Wilcoxon signed-rank tests with Holm adjustment for multiple comparisons. Higher scores on the global health status/QoL and functional scales indicate better functioning or quality of life, whereas higher scores on symptom scales indicate greater symptom burden.
