# Supplementary material for: Health-Related Quality of Life and Mid-Term Oncological Outcomes After Subcostal Uniportal Robot-Assisted Lung Cancer Surgery: A Secondary Analysis of a Clinical Trial
Source: Cancers (Basel). 2026 Aug 25;18(17):2763. doi: 10.3390/cancers18172763 (PMC13565155; doi:10.3390/cancers18172763)
Supplement: Supplementary file 1 [file cancers-18-02763-s001.zip › 20260820SUPPLEMENTARY_TABLE_S1.pdf]

Supplementary Table S1. Inclusion and exclusion criteria

**Inclusion criteria**
1. Age between 20 and 75 years
2. Ability and willingness to provide informed consent
3. American Society of Anesthesiologists (ASA) score ≤ 3
4. Diagnosis of clinical stage I lung cancer, with a primary tumor diameter ≤ 4 cm and at least 2 cm from the origin of the associated lobar bronchus
5. Preoperative platelet count between 150 and 400 $\times 10^3/\mu L$
**Exclusion criteria**
1. Congestive heart failure (New York Heart Association [NYHA] functional classification > II)
2. Known bleeding or clotting disorder
3. Active therapeutic dose anticoagulation or anti-platelet medications at the time of operation
4. Receiving immunomodulatory or immunosuppressive therapy (e.g., transplanted patients, steroid requirement) within 30 days prior to the planned surgery
5. Pulmonary hypertension
6. Requirement of extended resection (e.g., chest wall, carina, major vessel, bilobectomy) and reconstruction (e.g., sleeve resection, bronchoplasty, angioplasty)
7. History of ipsilateral thoracic surgery or sternotomy
8. Presence of an uncontrolled illness within the 6 months leading up to the planned surgical procedure – including, but not limited to, ongoing or active infections, symptomatic congestive heart failure, unstable angina pectoris, cardiac arrhythmia, or any psychiatric illnesses or social circumstances that could potentially hinder compliance with the study’s requirements
9. Previous neoadjuvant medical and/or radiation therapy
10. Contraindication for general anesthesia or surgery
11. Life expectancy of less than 6 months
12. Anatomy determined intra-operatively to be unsuitable for minimally invasive surgery
13. Belonging to vulnerable populations (e.g., pregnant or breastfeeding women)
14. International normalized ratio (INR) greater than 1.4
15. Activated partial thromboplastin time (APTT) greater than 35 seconds
