# Supplementary material for: Health-Related Quality of Life and Mid-Term Oncological Outcomes After Subcostal Uniportal Robot-Assisted Lung Cancer Surgery: A Secondary Analysis of a Clinical Trial
Source: Cancers (Basel). 2026 Aug 25;18(17):2763. doi: 10.3390/cancers18172763 (PMC13565155; doi:10.3390/cancers18172763)
Supplement: Supplementary file 1 [file cancers-18-02763-s001.zip › 20260820SUPPLEMENTARY_TABLE_S2.pdf]

Supplementary Table S2. Activity-specific and episode-specific pain outcomes at 3, 6, and 12 months

| Variable | Measure | 3 months | 6 months | 12 months | Overall $p$ value |
|---|---|---|---|---|---|
| Pain at rest | Patients with NRS > 0 | 0 (0.0%) | 0 (0.0%) | 0 (0.0%) | — |
| | NRS, entire cohort | 0 (0 – 0) | 0 (0 – 0) | 0 (0 – 0) | — |
| Pain while walking | Patients with NRS > 0 | 1 (3.0%) | 0 (0.0%) | 0 (0.0%) | 0.368 |
| | NRS, entire cohort | 0 (0 – 1) | 0 (0 – 0) | 0 (0 – 0) | 0.368 |
| Pain during physical activity | Patients with NRS > 0 | 6 (18.2%) | 2 (6.1%) | 0 (0.0%) | 0.009 |
| | NRS, entire cohort | 0 (0 – 2) | 0 (0 – 1) | 0 (0 – 0) | 0.009 |
| Most recent worst pain | Patients with NRS > 0 | 11 (33.3%) | 7 (21.2%) | 2 (6.1%) | 0.004 |
| | NRS, entire cohort | 0 (0 – 2) | 0 (0 – 1) | 0 (0 – 1) | 0.004 |
| Any postsurgical pain (NRS > 0 on at least one item) | Patients with NRS > 0 | 11 (33.3%) | 7 (21.2%) | 2 (6.1%) | 0.004 |

Values are presented as median (range) or number (percentage). Baseline NRS data were not collected. "Any postsurgical pain" refers to an NRS score > 0 on at least one of the four pain items, following the ICD-11 definition (see Methods, Section 2.5). Binary repeated outcomes were compared using Cochran's Q test; NRS scores were compared using the Friedman test. When the overall test was significant, pairwise comparisons were performed using exact McNemar tests or Wilcoxon signed-rank tests, as appropriate, with Holm adjustment for multiple comparisons. Maximum NRS values across the four items in the entire cohort and among symptomatic patients are reported in Table 2.
